# Listeriosis Outbreaks in British Columbia, Canada, Caused by Soft Ripened Cheese Contaminated from Environmental Sources

**DOI:** 10.1155/2015/131623

**Published:** 2015-03-30

**Authors:** Lorraine McIntyre, Lynn Wilcott, Monika Naus

**Affiliations:** ^1^Environmental Health Services, BC Centre for Disease Control, 655 West 12th Avenue, Vancouver, BC, Canada V5Z 4R4; ^2^Immunization Programs and Vaccine Preventable Diseases Services, BC Centre for Disease Control, 655 West 12th Avenue, Vancouver, BC, Canada V5Z 4R4; ^3^School of Population and Public Health, University of British Columbia, Vancouver, BC, Canada V6T 1Z9

## Abstract

Soft ripened cheese (SRC) caused over 130 foodborne illnesses in British Columbia (BC), Canada, during two separate listeriosis outbreaks. Multiple agencies investigated the events that lead to cheese contamination with* Listeria monocytogenes (L.m.)*, an environmentally ubiquitous foodborne pathogen. In both outbreaks pasteurized milk and the pasteurization process were ruled out as sources of contamination. In outbreak A, environmental transmission of* L.m.* likely occurred from farm animals to personnel to culture solutions used during cheese production. In outbreak B, birds were identified as likely contaminating the dairy plant's water supply and cheese during the curd-washing step. Issues noted during outbreak A included the risks of operating a dairy plant in a farm environment, potential for transfer of* L.m.* from the farm environment to the plant via shared toilet facilities, failure to clean and sanitize culture spray bottles, and cross-contamination during cheese aging.* L.m.* contamination in outbreak B was traced to wild swallows defecating in the plant's open cistern water reservoir and a multibarrier failure in the water disinfection system. These outbreaks led to enhanced inspection and surveillance of cheese plants, test and release programs for all SRC manufactured in BC, improvements in plant design and prevention programs, and reduced listeriosis incidence.

## 1. Introduction

*Listeria* is an environmentally ubiquitous Gram positive bacterium found in soil and vegetation, sewage, water, animal feeds, and food processing environments [1]. The pathogenic species* L. monocytogenes* (*L.m.*) infects domestic animals (i.e., cattle, sheep, goats, horses, poultry) and has also been found in wild avians, fish, and shellfish [2]. Of the eight species,* L.m.* is most often associated with human illness, although* L. ivanovii*, common in ruminant infections, is occasionally associated with human infection as well [3]. In humans, 99% of listeriosis cases are contracted through the consumption of contaminated food [4]. Healthy individuals rarely seek medical care for listeriosis infections, as these are self-limited with gastroenteritis and/or mild flu-like symptoms. However, elderly persons (>65 years), immune compromised individuals, neonates, and pregnant women and their fetuses are more susceptible to invasive forms of listeriosis infections, which can lead to encephalitis, meningitis, septicemia, and/or spontaneous abortions during the last trimester of pregnancy. Mortality rates for invasive listeriosis typically range between 20% and 40% [5, 6].

Several categories of ready-to-eat (RTE) foods have been associated with listeriosis outbreaks including vegetables (corn, celery, coleslaw, sprouts/taco salad) [6–9]; fruits (cantaloupe) [10]; processed deli meats (beef, turkey, hog head cheese, hot-dogs, cooked ham, jellied pork, RTE sandwiches) [11–17]; seafoods (crab meat, cold-smoked trout, smoked mussels, shrimp) [6, 18–20]; unpasteurized dairy products (Mexican soft cheese, raw milk cheeses, on farm fresh cheese) [21–25]; pasteurized dairy products (butter, soft cheese, sour milk curd cheese, fluid milk) [26–32].

Control of* L.m.* in food processing and retail environments is particularly difficult, due to its high cold tolerance (i.e., growth at refrigeration temperatures as low as −1.5°C) and its ability to form environmentally stable biofilms resistant to sanitation [6, 33–35]. In particular, in dairy milk and cheese processing,* L.m.* contamination may occur during transfer of raw fluid milk into the processing facility, from inadequate pasteurization, and from postpasteurization contamination during one or more of the following steps: addition of culture, cheese, curd formation, cutting, stirring, washing, moulding, draining, pressing, brining, salting, ripening, and packaging [36]. It has also been demonstrated that pathogens can be transferred from dairy animals to dairy processing plants [23], although cross-contamination of milking areas can be minimized [37].* L.m.* contamination may also be introduced via poor employee hygiene, via poor plant design, via equipment malfunction, from other nondairy ingredients (spices, starter cultures, water), and from inadequate sanitation and pest control. Proximity to farm environments may also be a risk factor for the introduction of* L.m.* into dairy processing plants, as increased incidence of* L.m.* has been linked to dairy farms with poor hygienic practices [38].

In the province of British Columbia (BC), Canada, cheese manufacture is regulated under the Milk Industry Act and Milk Industry Standard Regulations [39]. In BC, soft ripened cheese (SRC) aged for less than 60 days is only permitted to be made from pasteurized milk, reducing the risk of pathogen contamination through raw milk, although one other province (Quebec) allows the manufacture of raw milk soft ripened cheese. Two programs are used to control* L.m.* and other hazards in BC dairy plants: prerequisite programs and a Hazard Analysis Critical Control Point (HACCP) program [40, 41]. An effective HACCP program includes monitoring and control of critical control points established for the dairy pasteurization and postpasteurization process steps listed above. Effective prerequisite programs, also known as good manufacturing practices (GMPs), control the other sources of contamination found within and outside the dairy processing plant, such as employee hygiene and sanitation. Inadequate identification of food safety hazards, poor control of the processing environment, and lapses in carrying out established procedures can lead to food safety failures, allowing contamination to occur during cheese manufacturing steps. Both HACCP and prerequisite programs/GMPs must be functional for the safe manufacture of cheese.

Unlike harder types of cheese, SRC can be more vulnerable to postpasteurization bacterial contamination and subsequent outgrowth due to low acidity and high moisture content. For example, a Camembert would have 70% moisture and pH range of 5.5 to 5.8, whereas a harder Cheddar cheese would have 42% moisture and pH of 5.45 [42, 43]. The motile* L.m*. readily grows and multiplies in the cheese substrate and can penetrate SRCs [44]. During the ripening stage, SRC is typically held at 10°C and undergoes increasing alkalinity caused by the growth of bacteria and moulds on the surface of the cheese rind [42]. After ripening, SRC is refrigerated up to three months creating favourable conditions for the growth of cold-tolerant* L.m.* [44].

Although in theory many critical control points and potential failures in dairy processing have been described during the production of SRC, very few outbreak investigations have successfully identified and described the route of contamination of SRC during postpasteurization processing steps. Previous outbreaks involving pasteurized SRC have shown issues with cross-contamination at retail and by food handlers [28, 45, 46]. This paper describes the investigation of two separate pasteurized SRC outbreaks that occurred in BC, Canada, in 2002 that led to the uncovering of two novel environmental transmission pathways affecting postpasteurization processing.

## 2. Materials and Methods

Two listeriosis outbreaks (A and B) occurred in 2002 in BC, one in February 2002 (Plant A) and one in September 2002 (Plant B). Each outbreak was investigated by three means: via epidemiologic methods, laboratory analyses of samples, and plant investigations. Public health responses, in the form of health advisories and recalls, occurred as required by the investigation findings.

### 2.1. Epidemiologic Investigations

A multiagency investigation was required for both outbreaks. The communicable disease department of the BC Centre for Disease Control (BCCDC) coordinated case finding and followup, as well as the necessary restaurant and retail inspections which involved multiple health authorities. In addition to passive identification of cases self-reporting illness to their physician or emergency department, active case identification occurred following release of public health advisories that described the cheese implicated in the outbreaks. The information collected from cases included case demographics (age and gender), symptoms and illness onset, a description of exposure history, and other possible food vehicles. Cases were defined as symptomatic individuals exposed to the implicated SRC in their food history within the exposure period (up to 70 days after ingestion of the SRC). Confirmed invasive cases were identified through isolation of* L.m.* from a normally sterile site (i.e., blood or cerebral spinal fluid (CSF)) and had a compatible* L.m.* serotype and pulsed field gel electrophoresis (PFGE) biotype to the SRC; clinical cases presented with febrile gastroenteritis (vomiting, diarrhoea, and abdominal cramps) with or without positive stool identification of* L.m.* Confirmed noninvasive clinical cases with* L.m.* positive stool had a compatible* L.m.* serotype and PFGE biotype to the SRC.

### 2.2. Laboratory Investigations

Clinical (i.e., stool, blood, CSF), food (i.e., cheese and ingredients), water, and environmental samples (i.e., swabs from the plant, soil samples, animal faecal samples, compost, animal bedding, swabs from the farm environment and others) were tested according to standard culture methods. Briefly, any bacterial smears from sterile sites of suspected cases were referred to the provincial diagnostic laboratory for confirmation if they showed growth of* L.m.* on blood agar plates. Stool samples were screened for a variety of bacterial pathogens on respective selective enrichment agars:* L.m.*, other Gram positive pathogens (*Bacillus*,* Staphylococcus*), and routine enteric gram negative pathogens (*Salmonella*,* Shigella*,* Escherichia coli*,* Yersinia*,* Aeromonas*, and* Campylobacter*). In addition, screening for norovirus was conducted using reverse-transcription polymerase chain reaction [47]. Swabs of stool were cultured for* Listeria* via cold enrichment in* Listeria* enrichment broth for a minimum of 24 h followed by streaking bacteria onto selective agars of PALCAM, Oxford, and LPM (lithium chloride phenylethanol moxalactam) and incubation at 30°C for up to 48 h. Food, water, and environmental samples were assayed according to Health Canada's established procedures for* Listeria* in foods and environmental samples (MFHPB-30) and others [48–52]. Positive isolates were further characterized by serotyping and PFGE by the BCCDC Public Health Microbiology & Reference Laboratory and the National Microbiology Laboratory, as described elsewhere [53, 54]. In addition to* Listeria* testing, standard plate counts (SPC) and phosphatase tests were performed on raw milk samples; water samples were tested for heterotrophic plate counts (HPC) and total coliforms (TC). Additional testing of water colour and ultraviolet (UV) light absorbance and transmittance was conducted during the investigation of plant B [55, 56].

### 2.3. Plant Investigations

On-site inspections of each implicated dairy processing plant were conducted. Types and quantities of cheese manufactured at the plants from various milk sources were assessed for contamination, processing failure risks, and adherence to HACCP, prerequisite, programs and GMPs. Inspection focused on (1) raw milk quality and handling, (2) pasteurization effectiveness, (3) pasteurization procedures, (4) pasteurization equipment, and the likelihood of postpasteurization contamination from (5) raw milk, (6) the plant environment, (7) ingredients, and (8) personnel. Inspections included owner and operator interviews, review of on-site records and procedures, direct observation of processes involved during SRC production, testing of the dairy environment, testing of dairy ingredients (including water), and finished product sampling at the dairy processing plants. Further site inspections were conducted based on preliminary laboratory findings, and based on hypotheses that arose during the investigation.

## 3. Results

### 3.1. Epidemiologic Results

**Figure 1:**
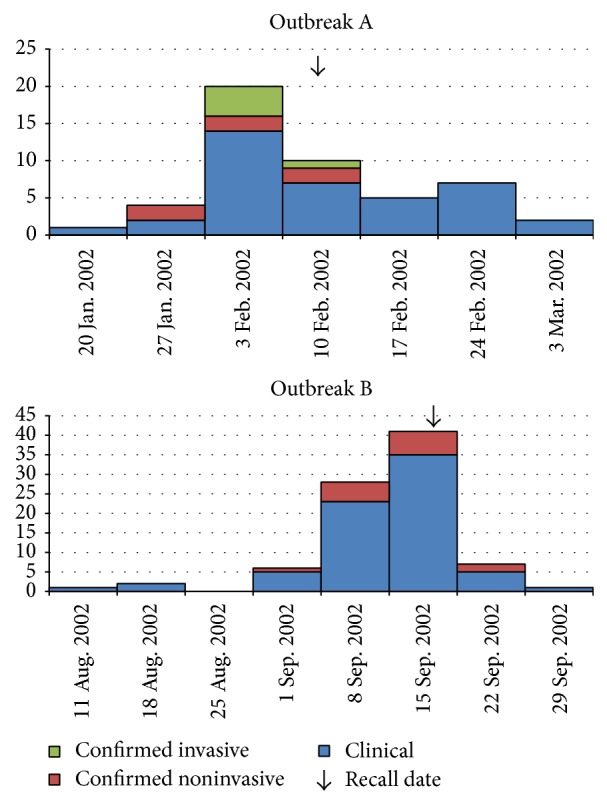
Epidemiological curves of weekly illness onsets for confirmed and clinical listeriosis in 2002 outbreaks.

In the first of the two outbreaks, two initial cases of bacterial meningitis caused by* L.m*. with onset of illness on February 3 and 7, 2002, respectively, provided a history of consumption of cheese produced by plant A and sold in a large farmers' market in Vancouver held on February 1–3, 2002. A Canadian Food Inspection Agency recall of all cheese manufactured in plant A was initiated on February 13, 2002. Trace-back activities revealed that 14 cheese varieties from plant A were sold to 20 different restaurant and retail premises on Vancouver Island and in Vancouver in the weeks prior to the outbreak (outbreak A). A total of 48 illnesses were linked to this outbreak: 43 cases with febrile gastroenteritis, three meningitis cases, and two cases of bacteremia in pregnancy (Table 1, Figure 1). The majority of illnesses were in females (64%), with ages ranging from four to 85 years (median 49 years). The median incubation period was seven days (range 1–33 days). The* L.m.* strain detected in food and clinical samples in this outbreak was serotyped as 4b, with PFGE pattern LMAAI.0140 (*Apa* I) and LMACI.0023 (*Asc* I). This* L.m.* strain was detected in five invasive (sterile site sample) and six noninvasive (stool sample) cases.

In the second outbreak, cheeses produced by plant B were sold at a farmers' market on Vancouver Island on September 6, 2002, and linked to a cluster of five illnesses within a family with rapid onset (<24 hrs) of severe febrile diarrhoea requiring hospitalization. An investigation began on September 18, one day following notification and receipt of food and clinical samples, and implicated cheese was recalled on September 19. A total of 86 cases, all with febrile gastroenteritis, were linked to this outbreak, with the earliest case identified one month prior, on August 15, 2002 (Table 1, Figure 1). The majority of illnesses were in females (72%), with ages ranging from 14 to 76 years (median 46 years). The median incubation period was two days from product consumption to onset of symptoms. The* L.m.* strain detected in food and clinical samples in this outbreak was serotyped as 4b, with PFGE pattern LMAAI.0017 (*Apa* I) and LMACI.0082 (*Asc* I). This* L.m.* strain was detected in 14 noninvasive (stool sample) cases.

### 3.2. Food and Environmental Testing Results

Overall, 113 and 104 food and environmental samples were collected and tested for the presence of* Listeria* spp. during outbreak A and B investigations, respectively. Additional sampling of raw milk in both outbreaks did not detect* L.m.* in raw milk sources and bacterial SPC were below the provincial standard of 4.70 log_10_ CFU/mL [57]. Phosphatase tests on pasteurized milk and cheese products were also negative in both outbreaks (Table 2).

#### 3.2.1. Plant A Results

*L.m.* positive cheese types associated with illnesses in outbreak A included those made of cow's and goat's milk, specifically SRC, fresh curds, a hard cheddar cheese, and chevre. Within the positive SRC, two varieties of cow cheese (tommes—a bacterial-smear ripened soft cheese and camembert—a soft-mould ripened cheese) and two varieties of goat cheese (same types) with one or more brand name(s) for each variety were distributed to restaurant and retail premises. A cheddar cheese, chevre made with goat's milk, and fresh cheese curds made with cow's milk were associated with illness. Several varieties were made and/or packaged on different dates, with between 8 and 12 distinct lot codes, spanning several weeks. The majority of cheese recovered (64%, *n* = 25) tested positive for* L.m.*, and all samples matched the outbreak serotype and PFGE pattern. Bacterial counts of* L.m.* ranged from <2 to 9.4 log_10_ CFU/g (median counts 2.0 log_10_ CFU/g) (Table 2).

Overall,* L.m.* was detected in 17 (19.3%) of the 88 environmental samples collected and included samples of ingredients, plant surfaces (e.g., food contact surfaces where cheese was aged and nonfood contact surfaces, such as drains, air vents), and the grounds area around plant A, including the adjacent hobby farm. Within the plant, seven samples were* L.m.* positive (9.6%, *n* = 73), all from a cheese aging room. These samples included two culture solutions stored in spray bottles, the shelf for storage of the spray bottles, condensate from the aging room blower unit, and a plastic bin where cheese was aged.* L.m.* counts of 7.3 log_10_ CFU/mL and 2.9 log_10_ CFU/mL were detected in* Penicillium* and* Brevibacterium* spray culture solutions, respectively. Outside the plant, 13 samples collected were found positive for* L.m.* (87%, *n* = 15), including a grass walkway leading to the plant, pig, and chicken areas on the farm. Other* Listeria* spp. (*L. innocua*) were also detected in these areas (Table 3).

#### 3.2.2. Plant B Results

*L.m.* positive cheese types associated with illnesses in outbreak B were limited to two varieties: a semihard cheese (raclette) and two varieties of SRC. Three lot/date codes were implicated. Contamination rates in these types of cheese were also high (76%, *n* = 29), with counts of* L.m.* ranging from <2 to 9.0 log_10_ CFU/g (median counts 2.0 log_10_ CFU/g), all of which matched the outbreak serotype and PFGE pattern (Table 2). Single lots of two cheese varieties were likely contaminated by a single lot of one SRC variety. These* L.m.* positive types of cheese were packaged with the single lot of contaminated SRC side by side on a cheese cutting board for sale at retail.* L.m.* counts in these types of cheese ranged from <2 log_10_ to 4.78 log_10_ CFU per gram, demonstrating the ability of the surface* L.m.* in the contaminated SRC to transfer to other types of cheese and multiply rapidly.

Overall,* L.m.* was detected in 14 (18.7%) of the 75 environmental samples collected and included one hydrated ingredient sample, water samples in and outside of the plant, and a variety of farm samples. Within the plant,* Listeria* spp. (*L. seeligeri*) were detected in two (6.2%) of the ingredients used in cheese production in plant B, a water sample taken from inside the plant (post-UV water treatment), and a hydrated mould culture prepared on September 16. All other plant surfaces (*n* = 22) and ingredients (*n* = 30) were negative for* Listeria* spp. Outside the plant and on the dairy farm, several different species of* Listeria* were detected, including* L.m.*,* L. seeligeri*,* L. innocua*, and* L. ivanovii* (Table 3).* L.m.* isolates recovered from a cistern pipe, a rag soaked with water in the milking house, and a swallow's nest sample matched the outbreak* L.m.* strain PFGE profile. HPC and TC results in cistern water were 2.01 log_10_ CFU per mL (HPC) and 1.0 log_10_ CFU per 100 mL (TC) and in post-UV water, 2.47 log_10_ CFU per mL (HPC) and 0.30 log_10_ CFU per 100 mL (TC).

### 3.3. Investigation Results

A review of the raw milk sources, pasteurization procedures, records, and equipment in both plants A and B did not reveal any obvious food safety hazards that could lead to* L.m.* contamination of finished products (Table 1). Both plants appeared clean and well maintained. The detailed investigation of plant A, however, did reveal potential issues with ingredients, equipment sharing between raw and RTE food areas, and the proximity of the farm animals to the plant. The detailed investigation of plant B suggested issues with the water supply, which were then further investigated.

#### 3.3.1. Plant A Investigation

In plant A, temperature records for raw milk receipts ranged from 0.8°C to 5.6°C, with acidity levels within normal range (pH 6.6 to 6.8). Once a week, 400 litres of raw goat's milk from a licensed dairy farm was used to make ~40 kg of cheese, and once every two weeks, 400 litres of raw cow's milk from the provincial dairy pool was used to make ~40 kg of cheese. All raw milk was vat pasteurized at 63.3°C for a minimum time of 30 minutes. Phosphatase tests of 11 different cheese types and production dates were negative, confirming that the milk used to make the cheese was properly pasteurized. The pasteurization equipment was tested to verify the accuracy of thermometers and timing clock. The integrity of vat jackets and the integrity of vat pasteurizer outlet protection valves were examined to ensure the absence of leakage. Raw milk cross-contamination into pasteurized milk was assessed during transfer of pasteurized milk to cheese vats. The potential for cross-contamination via operator (hands or clothing), equipment (used for both raw and pasteurized milk), and splashing was also evaluated. The operator demonstrated good understanding of the risks of cross-contamination, and hand/apron sanitizing was frequent. One piece of equipment, the pH meter, was found to be shared by raw and pasteurized milk sources. The probe was rinsed but not sanitized between testing of raw milk and pasteurized cheese curds. Testing of pH probe buffer solutions did not detect* L.m.* The probability of the pH probe and/or the pH probe buffer as a source of cross-contamination was assessed as unlikely (neutral investigation finding noted in Table 1). All areas of the dairy processing plant not in contact with contaminated cheeses or spray culture solution bottles were negative for* L.m.* In addition,* L.m.* and other* Listeria* spp. were detected in the environment outside plant A. This suggested a likely* L.m.* dissemination from the farm environment to the dairy processing plant, further supported by the finding that toilet facilities were shared between farm workers and dairy plant employees. However, a direct link to the farm through an animal vector could not be definitely established, as farm samples were collected several weeks into the investigation and it was discovered that during this time the operator had fed the recalled* L.m.* contaminated cheese to farm animals (i.e., pigs, housed next door to free-range chickens).

The interior of the dairy processing plant environment appeared clean, sanitary, and well maintained based on a visual inspection. Approved food grade sanitizers designed for use in a food processing environment were correctly employed, verified by a review of the sanitation records. Environmental sampling of the plant interior revealed only five of 40 (12.5%) swabs positive for* L.m.*, all from within the aging room (Table 2). Unacceptable investigation findings were found in the handling of two spray cultures in the aging room. A* Penicillium* mould culture solution used to spray the outer surface of the camembert soft mould-ripened cheese, and a* Brevibacterium* culture solution used to spray the outer surface of tommes bacterial smear-ripened cheese for fermentation were used for flavor and creation of a consistent rind on the outer surfaces of the cheeses. Preparation of solutions required rehydrating of freeze dried commercial culture that was added to boiled and cooled water containing 3% salt. The* Brevibacterium* culture solution also contained one part of commercial beer. Hydrated cultures were then stored in plastic spray bottles on a shelf in the aging room. However, these bottles were not regularly washed or sanitized. Further, new solution was added to the existing solution so that older solutions were not emptied out when bottles were refilled. The operator had typically stored the spray culture solution bottles in a 4°C refrigerator. However, this refrigerator malfunctioned three months prior to the outbreak, and the bottles were subsequently stored on a shelf in the aging room (kept at 10°C). The operator could not recall when the bottles had been last emptied, washed, and sanitized, describing this as occurring several weeks or months prior. Further, a worker occasionally prepared these solutions who had not received any training in dairy plant processes. As not all personnel working in the dairy processing plant were trained or licensed as dairy plant process workers, unsanitary handling of ingredients and equipment and unsatisfactory hygiene practices were also considered plausible causes of cross-contamination within the plant. Dairy processing plant entry access was also reviewed. Before personnel proceeded into the plant, entry was controlled by having personnel change clothes in a designated changing room area, put on dedicated plant shoes, and wash and sanitize hands in a sink in an adjacent toilet. However, the sink and toilet were shared with nondairy plant workers engaged in activities on the hobby farm, which included animals.

#### 3.3.2. Plant B Investigation

In plant B, records for August 16 indicated that the raw milk used to make the implicated batch of cheese was at 2.2°C prior to pasteurization. The pH of the milk was normal (pH = 6.8). SPC tests of the raw milk were performed twice per month, with a previous year annual average of 3.09 log_10_ CFU/mL. The raw cow's milk was supplied from the dairy processing plant's own licensed dairy farm located on the same site, and a daily production yield of 750 L of cow's milk was processed into several varieties of raw milk and pasteurized milk cheeses three times per week. Similar to the investigation of plant A, no issues were found with the pasteurization equipment or with the pasteurization method. Milk was vat pasteurized above the minimum pasteurization time and temperature to 65°C for 31 minutes. Further, no issues were identified to indicate any postpasteurization contamination of milk from raw milk (splashing or entering). The operator was also aware of potential cross-contamination issues. Raw milk and pasteurized milk cheese were not produced on the same day, and cleaning and sanitizing were performed at the end and beginning of each production. One of two dairy processing plant workers was responsible for milking cows, and the work duties, habits, and sanitary procedures of this worker were assessed as satisfactory. Clothing specific to the milking operation was put on over street clothes in the milk house. When called to work at the plant, the worker first washed hair and hands in the dairy farm milking house, removed clothing, and put on clean street clothes. At the plant office, street clothes were removed, and clean dairy clothes were put on before entering the dairy processing plant. Upon entering, rubber boots, apron, and hair net were worn (all used exclusively in the plant); then hands were washed and sanitized before proceeding into the processing room. A visual inspection of the processing areas did not reveal any deficiencies in the cleaning and sanitation program. These inspection findings were supported by environmental sampling, with none of the 22 swabs of food contact and nonfood contact surfaces within the plant testing positive for* Listeria* spp.

However, the ingredients used to manufacture cheese were not acceptable. Several ingredients were added to milk or cheese postpasteurization including freeze dried starter cultures, vegetable rennet, salt, natamycin, calcium chloride, annatto coloring, and hot water (66°C) to wash curds. During initial testing of 31 ingredients, only one ingredient (3.2%), a hydrated mould culture solution used on September 16 tested positive for* Listeria* spp. (*L. seeligeri*). Subsequent testing of all cultures and ingredients used in the hydrated solutions was negative, including an initial water test. An examination of the plant's production records revealed a critical anomaly: during the August 16 production of SRC, warm water at 45°C (a mixture of hot and cold water) was used to wash cheese curds, rather than the plants' normal procedure of using hot water at 66°C. Curd washing required the addition of 50 to 75 L of water to the cheese vat. This led to a hypothesis that the water used in the plant may have been the source of* L.m.*, and the investigation focused on the water supply. The initial test of in-plant water (100 mL was taken from the water hose in the plant used for curd washing) was negative for* L.m.* and other* Listeria* spp. When a larger sample of 1.5 L, rather than 100 mL, of tap water was examined on repeat sampling,* L. seeligeri* was found in the second test.

**Figure 2:**
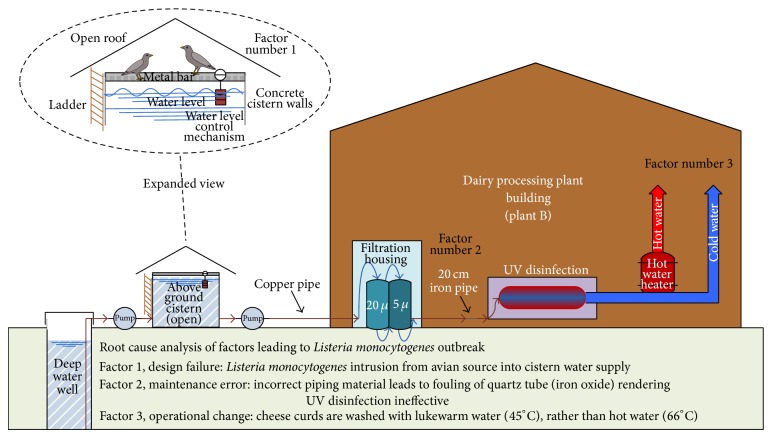
Schematic diagram of water supply system in dairy processing plant B.

Water for Plant B was supplied by a private deep well located several hundred metres away (Figure 2). Well water was pumped to an aboveground open concrete cistern located 150 m from the dairy processing plant. Although water samples from the cistern were negative for* Listeria* spp., total coliform and HPC results of cistern and post-UV treated water were unsatisfactory. The colour of the water in the cistern was elevated at 70 true colour units (TCU). However, an environmental swab of bird droppings on a pipe directly above the water surface in the cistern was positive and matched the PFGE profile of the outbreak* L.m.* strain. Other faecal and environmental samples in and around the farm revealed several* Listeria* spp., including matches to the outbreak strain in swabs taken from a rag regularly soaked with water in the milk house and in a barn swallow's nest (Table 3). From the cistern, water was pumped to the dairy processing plant and then through successive filters of 20 *μ*m and 5 *μ*m before passing through a UV water sterilizer into the plant. Further investigation of the water supply revealed a recently repaired section of piping supplying water to the UV sterilizer, where a 20 cm (8 inch) piece of iron pipe was spliced into the existing copper line. When the UV sterilizer was disassembled, a buildup of debris, suspected to be iron oxide, was found on the quartz sleeve that separated the water from the UV bulb. An engineer consultant calculated a UV transmittance of 47.5% based on the UV absorbance of the filtration unit (0.323 au) and on the elevated colour of the cistern water (with an unfouled quartz sleeve), a value considered extremely low. The combination of excessive cistern water colour and the fouled quartz sleeve would further lower the calculated UV transmittance, rendering the UV sterilizer ineffective.

## 4. Discussion and Conclusion

Postpasteurization contamination of SRC occurred in both outbreaks. Neither pasteurization failure nor contaminated milk supply were likely contributors to the outbreaks. Both dairy processing plants were visually very clean, and inspection observations found acceptable sanitation levels in the interior plant environments. These observations were supported by environmental swab tests of food contact and nonfood contact surfaces in the plants. In plant B, no swab samples were positive for* Listeria* spp., and in plant A only five surfaces, all in one room, were positive for* L.m.* However, inspections revealed that the external environments of both dairy processing plants were either neutral or unacceptable, observations also supported by test findings of high* Listeria* spp. and* L.m.* prevalence in areas outside the dairy processing plant and in the farm environment. Outbreak A events likely resulted from a GMP procedural failure arising from incorrect handling practices of culture spray solutions, while outbreak B was attributed to a multibarrier failure in the potable water supply to the plant. Contamination of the water held in the unprotected water cistern, failure in the UV water disinfection system, and subsequent addition of contaminated water in the curd washing step led to contamination of the SRC. Plant investigations and laboratory testing data identified barn swallows as the environmental reservoir and source of the* L.m.* in outbreak B. In outbreak A, while the source of the contamination was successfully traced to culture spray bottles and the plant's cheese aging room, whether the* L.m.* came from environmental sources outside the dairy processing plant could not be confirmed. Although a direct link could not be established, the most likely cross-contamination point was the shared toilet between farm and dairy processing plant workers, suggesting a potential for* L.m.* transmission from outside sources into the plant as* Listeria* is readily found in soil and farm environments. In both outbreaks, we posit that environmental transmission of very low numbers of* L.m.* was introduced during postpasteurization steps into SRC and other types of cheese, allowing growth of* L.m.* to very high numbers capable of causing illness.

As* L.m.* is a particularly cold-tolerant organism, very low numbers of* L.m.* may have initially contaminated the spray solutions in outbreak A. Over several months, we hypothesize that* L.m.* grew in the spray culture solution bottles, and when sprayed onto the cheeses, it further multiplied in the contaminated SRC. Processing of more than one variety of cheese likely resulted in cross-contamination among the types of cheese, with potential routes being from personnel handling the contaminated* L.m.* positive cheeses, from handling the spray culture bottles, and/or from* L.m.* on personnel clothing. A possible* L.m.* transmission route into the plant could be from individuals doing farm work, such as handling manure and garden dirt, leading to the contamination of shared toilet facilities and change room areas in the plant, though this theory is only speculative. While the outbreak* L.m.* strain was detected in several hobby farm samples, we could not confirm the animals or farm as the direct source, as* L.m.*-contaminated cheese recalled during the outbreak investigation had been fed to the pigs.

In outbreak B, the findings support a point-source contamination event that affected a single lot of SRC. The intensive investigation of the water source may not have occurred if the operator had not saved the September 16 hydrated culture solution. Following outbreak A and prior to outbreak B, the BCCDC had implemented a new directive to all operators to empty, clean, and sanitize hydrated culture solution bottles after use. However, out of prudence and concern, this solution was purposefully saved for subsequent testing by the operator of plant B upon notification of the recall and illness. A follow-up interview of the operator revealed that plant water normally used to wash curds was used to make the culture solution, instead of the recommended method of preparing the solution with boiled and cooled water.

Through a root cause analysis, it was revealed that the factors contributing to outbreak B included a design failure, a maintenance error, and an operational change (Figure 2). Individually, these factors would not have led to the cheese contamination event that resulted in a listeriosis outbreak. However, when combined, these factors created conditions that allowed* L.m.* into the water supply, its survival due to inadequate water treatment, and its transmission into the food during postpasteurization cheese processing. Focused investigation into the water supply revealed multiple issues: (1) the cistern was open to birds, and barn swallows were observed to sit on the metal bar directly above the surface of the water, drink from the cistern water supply, and defecate on the bar and into the cistern; (2) dried bird droppings were also observed on the upper lip of the cistern, and droppings were collected from the metal bar directly above the water located in the cistern; (3) the colour of water measured in the cistern was found to be elevated; and (4) an investigation of the UV disinfection system, once disassembled, revealed fouling of the quartz tube, likely from iron oxide, from the recent repair and splicing of a section of iron pipe into the water supply line. This resulted in minimal microbial reduction in water treated with this UV disinfection system. This assessment was supported by the HPC results, with 65% more bacteria found in the treated versus untreated (cistern) water supply.

Although it is not known how long the UV disinfection system was failing, hot water (66°C), normally used to wash the curds would likely have killed any* Listeria* organisms that had survived the faulty UV water sterilizer. The August 16 lot code of SRC is suspected to have been contaminated with* L.m.* from the in-house treated water supply. On this date, only warm water (45°C from a mixture of hot and cold water), not hot water, was used to wash the curds. While washing curds with warm water is a normal and acceptable practice in dairy processing plants, we suspect that addition of warm water to the curds allowed the introduction of viable* L.m.* into the cheese curd from the water supply. The original source of the* L.m.* in the water supply pointed to barn swallows as the outbreak strain of* L.m.* was found on the pipe in the cistern containing bird droppings, in a barn swallow's nest in the farm area, and on a water rag used in the milking house (Table 2).

Another interesting finding from outbreak B was the unusual illness presentation, with only noninvasive listeriosis cases observed.* L.m.* was detected in clinical samples of stool (faeces). In the year of the outbreak, 2002, neither invasive nor noninvasive listeriosis were nationally nor provincially notifiable diseases. After the two 2002 listeriosis outbreaks in BC, invasive listeriosis disease became a reportable condition in the province of BC [58]. Nationally, invasive listeriosis became a reportable condition in 2007, although noninvasive cases are still not reportable or tracked in Canada, including BC [58, 59]. We report here a very rare event where a noninvasive* L.m.* outbreak was discovered and for the first time reported in BC and Canada. Findings from the two BC outbreaks described here and other provincial outbreaks were shared with the federal authorities and collectively led to improvements in the Canadian listeriosis reference services through offering of enhanced and prompt PFGE testing, creation of standardized food histories, and recommendations for testing for* L.m.* in cases of noninvasive febrile gastroenteritis, when other pathogens were not detected in stool [60].

Immediate recommendations made by BC provincial authorities included a requirement for the two dairy processors involved in the outbreaks to test for* L.m.* before releasing their products for sale, and a new requirement for periodic industry funded testing of SRC products for* L.m.* from other provincial dairy processing plants. All dairy processing plants were required to submit current HACCP-based or equivalent food safety plans, provide product lists for their operations, demonstrate water used as an ingredient meets requirements for potability, and ensure that effective physical barriers exist between their plants and other agricultural uses. Plant B was also required to bring in water to be held in a closed containment system for processing. Further recommendations for dairy and public health inspectors were to conduct a review of all private water systems supplying potable water to dairy processing plants, include the management of ripening solutions under the HACCP procedures for plants, incorporate additional materials into the dairy worker course, regularly collect environmental swabs for* L.m.* testing, and conduct annual auditing of SRC products during inspections.

Inspections of dairy processing plants did lead to industry improvements and a reduction in the numbers of listeriosis cases in the years following the 2002 outbreaks. In a 2009 survey of dairy, meat, and fish processors in BC, no dairy products nor processing food contact surfaces in dairy processing plants were found to contain* L.m.* or other* Listeria* spp. [61]. No illnesses linked to SRC produced in dairy processing plants under provincial inspection authority have been detected since 2002. Routine inspections, however, have occasionally detected* L.m.* in cheese and environmental swabs. Noncompliant food and environmental swab test results and noncompliant observations during inspections have led to both product recalls and incremental improvements in dairy processing plants when deficiencies noted on inspections are addressed (unpublished data). We believe that these interventions, which arose from direct inspection observations and sample testing, have contributed to the prevention of illness and are necessary for public health.

In summary, investigations of foodborne outbreaks can be complex, requiring multiagency support, and extensive on-site inspection before the root cause of pathogen contamination of manufactured foods can be established. Specifically, in the outbreaks reported here, environmental sampling assisted in focusing on the inspections, generating hypotheses, and formulating the questions asked of plant operators during follow-up inspection interviews.* L.m.* transmission into cheese was uncovered during subsequent operator interviews and investigations. The complexity of the investigations required coordinated response from multiple experts, including dairy and health inspectors, epidemiologists, engineers, laboratory technologists, agrologists, and physicians.

Inspectors and regulators responsible for oversight of manufacturing processes require detailed systems knowledge to understand where errors can occur. Many regulatory agencies are moving towards outcome based guidance, reliance on inspection of records, and compliance with record keeping. In outbreak B, there should not have been an outbreak when the dairy worker washed the curds with lukewarm water. The issue was that the water should have been potable, and it was not. This dairy had a secondary UV water disinfection system, supported with monitoring records to show that the system had been operating normally and had been maintained as required by the system manufacturer. From a regulatory and records perspective, the dairy was in full compliance. The problem lay in the source water contamination (cistern was open to animals) compounded by a recent improper repair to the water line. The multiple factor failures illustrate how events can lead to illness, despite compliance with regulations and despite good records. A surface examination of records would not have revealed these problems. We are concerned that with regulatory agencies now moving towards a model of records inspection concomitant with a reduced inspection frequency in manufacturing settings could potentially lead to missed opportunities for detecting and correcting errors that are often found during physical and process inspections.

In addition, findings from these listeriosis outbreaks demonstrate the importance of adhering to strict processing procedures to minimize the survival and spread of* L.m.* during postpasteurization product handling and that environmental transmission of* L.m.* into foods can occur from wild animal sources. Further, considering that products implicated in the outbreaks were prepared from pasteurized milk and that these products may be erroneously considered safe for consumption by populations vulnerable for listeriosis [30], we recommend that pregnant women and immunocompromised and elderly populations >65 years old should avoid the consumption of pasteurized or unpasteurized SRC. This recommendation is consistent with relative susceptibility risk of these populations to listeriosis and with the advice given by the federal government of Canada [62, 63]. Ongoing vigilance from food manufacturers and public health inspectors are necessary to limit opportunities for harmful bacteria to enter the food supply. As* Listeria* is ubiquitous in the environment, special precautions are recommended for dairy processing plants and other food processing plants located adjacent to farms and wildlife animals.

## Figures and Tables

**Table 1:** Summary of outbreak findings.

	Outbreak A	Outbreak B
Illnesses		
Total number of illnesses	49	86
First reported illness	February 3, 2002	August 15, 2002
Organism identified	*L.m. *	*L.m. *
Serotype	4b	4b
PFGE designations—Apa I	LMAAI.0140	LMAAI.0017
—Asc I	LMACI.0023	LMACI.0082
Number of noninvasive (clinical) cases		
Febrile gastroenteritis (stool+)	44 (6)	86 (14)
Number of invasive (confirmed) cases		
Meningitis	3	0
Bacteremia in pregnancy	2	0
Demographics		
Age range in years (median)	4 to 85 (49)	14 to 76 (46)
% Female	64	72
Clinical findings		
Incubation period in days (median)	1 to 33 (7)	0.5 to 28 (2)
Symptoms reported (%)		
Fatigue	51	62
Myalgia	46	55
Chills	38	0
Night sweats	24	0
Bone pain	19	0
Abdominal pain	8	54
Cheese^1^ prepared at plant		
Number of cheese types produced	14	10+
Bacterial smear surface soft ripened cheese	**Y** **e** **s** ^2∗^	Yes
Chevre (goat milk soft cheese)	**Y** **e** **s** ^∗^	No
Curds (e.g., cheddar)	**Y** **e** **s** ^∗^	Yes
Feta cheese	Yes	Yes
Soft cheese (e.g., fromage frais)	Yes	Yes
Hard cheese (e.g., cheddar cheese)	**Y** **e** **s** ^∗^	Yes
Semihard cheese (e.g., raclette)	No	**Y** **e** **s** ^∗^
Soft mould ripened cheese	**Y** **e** **s** ^∗^	**Y** **e** **s** ^∗^
Investigation findings (Acceptable/neutral/unacceptable)		
Raw milk quality and handling	Acceptable	Acceptable
Pasteurization effectiveness/procedures	Acceptable	Acceptable
Pasteurization equipment	Acceptable	Acceptable
Postpasteurization—raw milk contamination	**Neutral**	Acceptable
Postpasteurization—interior plant environment	Acceptable	Acceptable
Postpasteurization—ingredients	**Unacceptable**	**Unacceptable**
Postpasteurization—personnel	**Unacceptable**	Acceptable
External environment	**Neutral**	**Unacceptable**

^1^Cheese types made with cow *or *goat milk unless specified; ^2^cheese products linked to illness are indicated in bold with^∗^; *L.m.*, *Listeria monocyotogenes*.

**Table 2:** Results of lab tests in milk, cheese, and environmental samples.

	Outbreak A	Outbreak B
Milk samples		
Raw milk SPC (log_10_ CFU/mL)		
(1) Government dairy pool	3.90	n/a
(2) Local farm—cow	n/a	3.00
(3) Local farm—goat	4.43	n/a
Raw cow milk *L.m. *	Absent	Absent
Raw cow milk pH	NT	6.8
Pasteurized cow milk phosphatase	Negative	Negative
Cheese samples		
Number of cheese samples *L.m*. + (tested)	16 (25)	22 (29)
Number of varieties + (number of lots+)	8 (12)	3 (3)
*L.m*. counts (median log_10_ CFU/g)	2.0	2.0
*L.m*. counts (range log_10_ CFU/g)	<2 to 9.4	<2 to 9.0
Environmental samples		
In the plant—ingredients		
Number of *L. *spp. + (tested)	2 (33)	2 (32)
In the plant—surfaces		
FCS number of *L *spp. + (tested)	3 (17)	0 (5)
NFCS number of *L *spp. + (tested)	2 (23)	0 (17)
Outside the plant		
Number of *L *spp. + (tested)	1 (1)	4 (7)
On the hobby or dairy farm		
Number of *L *spp. + (tested)	12 (14)	6 (14)
Number of environmental *L.m*. + (tested)	17 (88)	14 (75)

SPC, standard plate count; NT, not tested; n/a, not applicable; *L.m.*, *Listeria monocytogenes*; *L. *spp., *Listeria *species (*L. innocua, L. ivanovii *or *L. seeligeri*); FCS, food contact surface; NFCS, nonfood contact surface.

**Table 3:** *Listeria *spp. detailed results from investigations.

Sample description	*Listeria *spp.	Matched to cheese *L.m. *strain?
Outbreak A samples		
*Brevibacterium *culture spray solution	*L. monocytogenes *	Yes
*Penicillium* mould spray solution	*L. monocytogenes *	Yes
Aging room shelf (where spray bottles stored)	*L. monocytogenes *	NT^1^
Aging room—inside plastic aging containers (3 samples)	*L. monocytogenes *	Yes
Aging room—condensate from blower unit	*L. monocytogenes *	Yes
Whey trench outside	*L. monocytogenes *	NT
Grass beside walkway	*L. monocytogenes *	NT
Pig garden—poo area	*L. monocytogenes *	NT
Pig garden—wet bedding	*L. innocua *	
Pig garden—compost pile	*L. monocytogenes *and *L. innocua *	NT
Pig garden—whey tank area	*L. innocua *	
Dog run	*L. monocytogenes *	NT
Pig pen water	*L. innocua *	
Pig pen dirt	*L. monocytogenes *	Yes
Pig pen bedding	*L. monocytogenes *	Yes
Chicken coop floor—dirt	*L. monocytogenes *	NT
Chicken run—old flooring	*L. monocytogenes *	NT
Chicken run walkway	*L. monocytogenes *	NT
Outbreak B samples		
Hydrated mould culture	*L. seeligeri *	
Finished water (UV treated/filtered from inside plant)	*L. seeligeri *	
Cistern pipe	*L. monocytogenes *	Yes
Pond water	*L. seeligeri *	
Lagoon water	*L. innocua *	
Sewage water	*L. monocytogenes *	NT
Cow feces	*L. innocua *	
Cow feed greens	*L. monocytogenes *	No
Water/rag in milk house	*L. monocytogenes *	Yes
Swallow nest	*L. monocytogenes *	Yes
Chicken feces	*L. ivanovii *	
Pheasant feces	*L. monocytogenes *	No

^1^NT, not tested.
